# Gallbladder cancer: current and future treatment options

**DOI:** 10.3389/fphar.2023.1183619

**Published:** 2023-05-11

**Authors:** Yanzhao Zhou, Kun Yuan, Yi Yang, Zemin Ji, Dezheng Zhou, Jingzhong Ouyang, Zhengzheng Wang, Fuqiang Wang, Chang Liu, Qingjun Li, Qi Zhang, Qiang Li, Xiao Shan, Jinxue Zhou

**Affiliations:** ^1^ Department of Hepatobiliary and Pancreatic Surgery, The Affiliated Cancer Hospital of Zhengzhou University, Henan Cancer Hospital, Zhengzhou, China; ^2^ Department of Hepatobiliary Cancer, Liver Cancer Research Center, National Clinical Research Center for Cancer, Key Laboratory of Cancer Prevention and Therapy, Tianjin’s Clinical Research Center for Cancer, Tianjin Medical University Cancer Institute and Hospital, Tianjin, China; ^3^ Department of Hepatobiliary Surgery, National Cancer Center/National Clinical Research Center for Cancer/Cancer Hospital, Chinese Academy of Medical Sciences and Peking Union Medical College, Beijing, China

**Keywords:** gallbladder cancer, clinical stage, adjuvant therapy, targeted therapy, immunotherapy

## Abstract

Surgery remains the preferred treatment option for early-stage gallbladder cancer (GBC). According to the anatomical position of the primary tumor, accurate preoperative stage and strict control of surgical indications, appropriate surgical strategies are selected to achieve the optimal surgical effect. However, most patients have already been at the locally advanced stage or the tumor has metastasized at the initial diagnosis. The postoperative recurrence rate and 5-year survival rate remain unsatisfactory even after radical resection for gallbladder cancer. Hence, there is an urgent need for more treatment options, such as neoadjuvant therapy, postoperative adjuvant therapy and first-line and second-line treatments of local progression and metastasis, in the whole-course treatment management of gallbladder cancer patients. In recent years, the application of molecular targeted drugs and immunotherapy has brought greater hope and broader prospects for the treatment of gallbladder cancer, but their effects in improving the prognosis of patients still lack sufficient evidence-based medicine evidence, so many problems should be addressed by further research. Based on the latest progress in gallbladder cancer research, this review systematically analyzes the treatment trends of gallbladder cancer.

## 1 Introduction

Gallbladder cancer (GBC) takes up approximately 2/3 of all malignancies of the biliary tract, which is the most common type. Because of the special anatomical location of GBC and the insidious and non-specific symptoms of GBC, most patients have already been at an advanced stage at diagnosis, and only about 25% of them have the opportunity for surgery. Moreover, GBC may recur in about 60%–70% of patients after surgery, so the prognosis of GBC remains unsatisfactory, and the 5-year survival rate of these patients is only about 5%–15% (Hundal and Shaffer, 2014; Sharma et al., 2017; Roa et al., 2022). At present, a comprehensive treatment model dominated by radical surgical resection is still adopted for the treatment of GBC. Gemcitabine and platinum-based chemotherapies have been the main treatment modality for unresectable, locally advanced, and metastatic gallbladder cancer (Goetze, 2015; Roa et al., 2022). In recent years, based on the emergence of a new generation of sequencing technology, the therapeutic strategies of GBC are constantly updated, and the use of GBC molecular profiling promotes the development and subsequent clinical application of novel targeted and immunotherapeutic drugs (Javle et al., 2014; Nakamura et al., 2015). The epidermal growth factor receptor (EGFR), fibroblast growth factor (FGFR), human epidermal growth factor receptor 2 (HER2), and PD-1/PD-L1 have all been used successfully as therapeutic targets in clinical trials among the numerous genomic alterations identified in GBC (Ho et al., 2022), and this has resulted in an evolving paradigm for the treatment of gallbladder cancer. In the following sections of this review, the latest treatment trends of GBC will be systematically demonstrated from five aspects, i.e., the surgical treatment of GBC, neoadjuvant therapy, postoperative adjuvant therapy, treatment of unresectable advanced GBC, and targeted therapy and immunotherapy.

## 2 Surgical treatment of GBC

**FIGURE 1:**
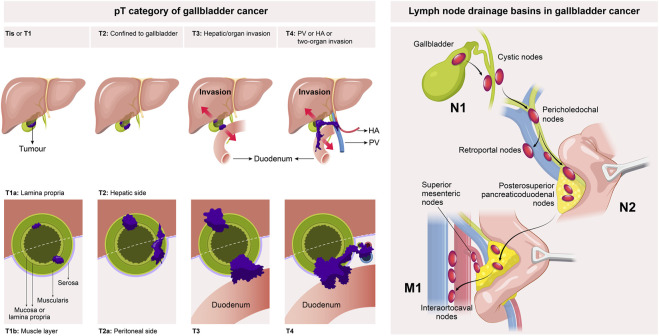
AJCC 8th edition TNM staging system for gallbladder cancer.

**FIGURE 2:**
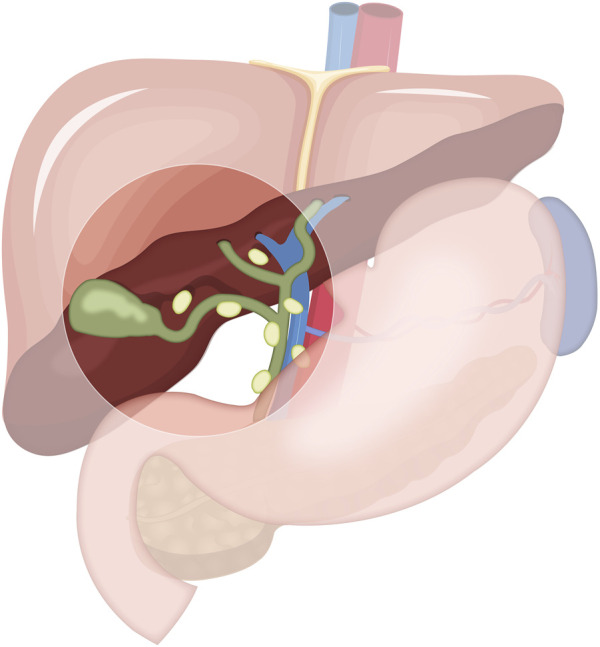
A schematic of radical cholecystectomy including cholecystectomy, segment IVB and V resection, and portal lymphadenectomy.

Surgical treatment is the first-line therapy for early-stage GBC. To achieve long-term survival, radical resection is the only treatment option for GBC patients, and preoperative confirmation of the clinical stage of GBC is a vital precondition for the standard radical resection of GBC. At present, GBC is clinically staged mainly using the tumor-node-metastasis (TNM) staging system recommended by the American Joint Committee on Cancer (AJCC) (Table 1) (Amin et al., 2017). As per the AJCC 8th edition staging system, the clinical stage of GBC was evaluated based on the depth of the primary tumor infiltrating the gallbladder wall and liver tissue (T1-3), the number of regionally metastasizing lymph nodes (N1-2), and the presence or absence of distant metastasis (M0-1) and large blood vessel invasion (T4) (Figure 1), in which the T stage determined the extent of hepatectomy for GBC, i.e., simple resection, standard radical resection, or extended radical resection (Figure 2).

**TABLE 1:** AJCC 8th edition TNM staging system for gallbladder cancer.

T stage	T criteria
TX	Primary tumor cannot be assessed
T0	No evidence of primary tumor
Tis	Carcinoma *in situ*
T1	Tumor invades the lamina propria or muscular layer
T1a	Tumor invades lamina propria
T1b	Tumor invades muscle layer
T2	Tumor invades the perimuscular connective tissue on the peritoneal side, without involvement of the serosa (visceral peritoneum) or tumor invades the perimuscular connective tissue on the hepatic side, with no extension into the liver
T2a	Tumor invades the perimuscularr connective tissue on the peritoneal side, without involvement of the serosa (visceral peritoneum)
T2b	Tumor invades the perimuscular connective tissue on the hepatic side, with no extension into the liver
T3	Tumor perforates the serosa (visceral peritoneum) and/or directly invades the liver and/or other adjacent organ or structure, such as the stomach, duodenum, colon, pancreas, omentum, or extrahepatic bile ducts
T4	Tumor invades main portal vein or hepatic artery or invades two or more extrahepatic organs or structures
N stage	Regional lymph nodes
NX	Regional lymph nodes cannot be assessed
N0	No regional lymph node metastasis
N1	Metastasis to one to three regional lymph nodes
N2	Metastasis to four or more regional lymph nodes
M stage	Distant metastasis
M0	No distant metastasis
M1	Distant metastasis

### 2.1 Tis/T1 stage

Tis/T1a GBC only invades the lamina propria of the gallbladder mucosa, which can be treated by simple resection, with a 5-year survival rate of 100% (Shirai et al., 1992; You et al., 2008). However, there has been an argument over the treatment of T1b GBC. According to previous studies, lymph node metastasis occurred in 15%–21% of T1b GBC patients, which was much higher than that in T1a patients (Ogura et al., 1991; Vo et al., 2019; Goel et al., 2022). The postoperative recurrence rate of T1b GBC patients was significantly higher than that of T1a GBC patients (9% vs. 1%, *p* < 0.01). In addition, the recurrence rate of T1b GBC patients after simple resection was 12.5%, and that after extended cholecystectomy was 2.7% (Lee et al., 2011). Abramson et al. (2009) established a Markov decision model to evaluate patients’ life expectancy related to surgical strategies. The results revealed that the survival of T1b GBC patients after radical resection was about 3 years and 5 months longer than that of patients with simple resection (9.85 years vs. 6.42 years). Vo et al. (2019) analyzed T1b GBC patients in the National Cancer Data Base from 2004 to 2012, and found that the 5-year overall survival (OS) rate would be reduced from 64.4% to 48.3% if the patients only received simple resection. Hence, it is highly recommended to perform the same radical operation as T2, especially regional lymphadenectomy, for T1b GBC patients (Fetzner et al., 2011; Vo et al., 2019; Goel et al., 2022). Xu et al. (2020) conducted specific research on the optimal number of regional lymph nodes that should be dissected and found that patients undergoing simple resection and lymphadenectomy (>5 lymph nodes) had a higher overall survival (OS) rate than those not undergoing regional lymphadenectomy. GBC exhibits a high degree of malignancy and a high rate of lymph node metastasis; regional lymphadenectomy is accompanied by a low incidence of complications and a low mortality rate, and determining the status of lymph node metastasis contributes to the formulation of postoperative adjuvant therapy for patients (Goel et al., 2022). Therefore, it is suggested that T1b GBC patients with physical tolerance should undergo radical resection of GBC (the resection can be divided into regional lymphadenectomy and wedge resection of the liver at 2 cm away from gallbladder bed according to the resection extent), and it should be confirmed that the resection margins of the cystic duct and liver tissue are negative, so as to achieve more accurate tumor staging, guide subsequent adjuvant therapy and evaluate the survival of patients.

### 2.2 T2 stage

According to the AJCC 8th edition TNM staging system, the T2 stage of GBC can be further divided into T2a stage (the tumor on the peritoneal side invades the perimuscular connective tissue without exceeding the serosa) and T2b stage (the tumor on the liver side invades the perimuscular connective tissue without exceeding the serosa) (Figure 1) (Amin et al., 2017). The prognosis of T2b GBC patients is worse than that of T2a GBC patients. Shindoh et al. (2015) enrolled 252 T2 GBC patients for research, and it was found that the 3-year and 5-year survival rates of T2a and T2b GBC patients undergoing radical resection largely differed from those undergoing simple resection (75.5% vs. 49.8%, *p* = 0.006, and 48.2% vs. 28.9%, *p* = 0.018). The 3-year and 5-year survival rates of T2b GBC patients were 52.1% and 42.6%, respectively, while those of T2a GBC patients were 73.7% and 64.7%, respectively, showing statistically significant differences (*p* = 0.0006). This result may be attributed to the higher residual rate of cancer cells in the gallbladder bed (18% vs. 0%, *p* = 0.001), higher lymph node metastasis rate (28% vs. 17%, *p* = 0.18) and more micrometastases in the adjacent liver parenchyma (33% vs. 6%, *p* < 0.001) in T2b GBC patients compared with those in T2a GBC patients. A recent meta-analysis also supports the above conclusion, which summarizes 15 retrospective studies (a total of 2,531 patients, including 1,332 T2a GBC patients and 1,199 T2b GBC patients). According to the results, compared with T2a GBC patients, T2b GBC patients have a worse prognosis and a higher risk of recurrence, and hepatectomy can prolong the OS of T2b GBC patients (Alrawashdeh et al., 2022). Toge et al. (2019) reviewed 81 T2 GBC patients and found that the lymph node metastasis rate of T2b GBC patients was higher than that of T2a GBC patients (46% vs. 20%, *p* = 0.028), but differences in the number of positive lymph nodes and anatomical metastasis distribution between the two groups were not statistically significant, so the extent of regional lymph node resection of T2a and T2b GBC did not need to be changed. Maruyama et al. (2019) evaluated the correlation between tumor location and peripheral nerve invasion. The results manifested that peripheral nerve invasion was more frequently detected on the liver side and proximal end, but less detected on the peritoneal side and distal end, indicating that extrahepatic bile duct resection may not be necessary for peritoneal and distal GBC. In regard to the extent of hepatectomy and lymphadenectomy for T2 GBC, it has been shown that there is no advantage in the prognosis of patients with anatomical hepatectomy (IVb and V segment resection) compared with that of patients with non-anatomical hepatectomy (gallbladder bed resection) (Horiguchi et al., 2013; Kwon et al., 2020). Japan’s Clinical Practice Guidelines for the Management of Biliary Tract Cancers 2019 tends to recommend non-anatomical wedge resection on the premise that R0 resection can be achieved, but a consensus on the width of wedge resection has not been reached (Nagino et al., 2021). The optimal extent of lymphadenectomy for radical resection of GBC remains uncertain, but whether lymph nodes are positive is an important predictor of survival after the radical resection of GBC, so at least six lymph nodes should be resected for correct and complete staging (Ito et al., 2011; Aloia et al., 2015). To sum up, the anatomical location of the tumor on the liver side differs from that on the peritoneal side, which leads to a significant difference in the prognosis of patients after resection. Hence, the molecular biological mechanism leading to the above differences should be explored in future research.

### 2.3 T3/4 stage

Patients with T3/4 GBC are in a locally advanced stage and should undergo extended radical resection, including vascular resection and reconstruction, extended right hemihepatectomy, extended lymphadenectomy, extrahepatic bile duct resection and even combined pancreaticoduodenectomy. The research results of D'Hondt et al. (2013) revealed that only 28.6% of T3 GBC patients could be treated by radical resection. Pilgrim et al. (2009) reported that if R0 resection could be achieved by surgical treatment in T3 GBC patients, the 5-year survival rate could still reach 63%–67%. Mizuno et al. (2019) proposed that hepatopancreatoduodenectomy (HPD) was a better surgical strategy for T3/4 GBC, and the median survival of patients undergoing HPD and those without HPD was 10 months and 6 months, respectively (*p* = 0.036). Further analysis of distant metastasis in subgroups revealed that the survival of M0 patients receiving HPD was longer (the median survival was 20 months), while that of M1 patients with or without HPD was shorter (the median survival was 6 months). It should be noted that the high incidence rate of postoperative complications and high mortality rate limit the wide application of HPD. Aoki et al. (2018) reported that the application of two-stage pancreaticojejunostomy in HPD could reduce the incidence rate of major complications such as pancreatic fistula after surgery, and the postoperative mortality rate was almost zero. The team also proposed that associating liver partition and portal vein ligation for staged hepatectomy (ALPPS) can be applied to prevent extensive hepatectomy-induced liver failure by promoting liver regeneration before HPD (Sakamoto et al., 2016). Sahara et al. (2020) also discussed the importance of lymphadenectomy by exploring the surgical indications of T4 GBC according to the therapeutic index (lymph node metastasis rate × 3-year OS). The results suggested that the effects of lymphadenectomy on GBC patients in stage T4 or those with CA19-9 ≥ 200 UI/mL are limited. Higuchi et al. (2020) investigated the adverse prognostic factors that affect the long-term postoperative efficacy of T3 and T4 GBC patients. It was found that there were ≥2 of the preoperative factors (invasion of the liver ≥5 mm, invasion of the left margin of the hepatoduodenal ligament or the whole area, and metastasis of ≥4 lymph nodes), indicating poor prognosis. In addition, it has been demonstrated by some research results that resection of T3/4 GBC is meaningful only when R0 resection was achieved (Birnbaum et al., 2014; D'Souza et al., 2020). Although surgical resection is valuable for prolonging the survival of T3/4 GBC patients to some extent, there is still an argument over surgical treatment due to the poor prognosis of locally advanced GBC resection as well as the high incidence rate of complications related to extensive resection and the high mortality rate. In addition, before the implementation of the extended resection, the possibility that the patient’s postoperative recovery time will lead to a decrease in the adjuvant chemotherapy rate and the poor quality of life must also be considered (D'Souza et al., 2020; Kuipers et al., 2021a; Welch et al., 2020; Shroff et al., 2019a). Therefore, the technical resectability is not equivalent to the biological resectability of the tumor, and the large proportions of patients receiving HPD suffered from lymph node metastasis and extensive hepatoduodenal ligament and pancreatic head invasion by the tumor. Hence, the indications of HPD deserve further discussion, and the clinical application in pancreatic cancer can provide a reference. It is urgent to establish clear concepts of “resectable,” “borderline resectable,” and “unresectable” GBC (Chaudhari et al., 2018; Mizuno et al., 2019). All these situations indicate that in addition to simple surgery, new treatment strategies are needed to improve the prognosis of patients. For patients with locally advanced GBC, such as T4 GBC, a treatment option involving multidisciplinary team cooperation should be selected to avoid excessive invasiveness, while neoadjuvant therapy may be beneficial for certain locally advanced patients by reducing the tumor stage to create radical surgery opportunities and reducing the risk of postoperative relapse.

## 3 Neoadjuvant therapy

Neoadjuvant therapy is gradually becoming a treatment option for GBC patients, which is expected to become a new treatment strategy for improving the prognosis of GBC patients. Reports have shown that this treatment strategy has allowed some patients with locally advanced unresectable GBC to undergo surgeries or improved the prognosis of patients with resectable GBC (Kato et al., 2013; Ozer et al., 2022). The expected advantages of neoadjuvant therapy include (Medin and Maithel, 2022): (Hundal and Shaffer, 2014) reducing the primary focus to improve the R0 resection rate, (Sharma et al., 2017), treating distant metastases that are difficult to detect by imaging examination, (Roa et al., 2022), avoiding the impact of postoperative complications on preoperative treatment, thereby improving the completion rate of adjuvant therapy, and (Goetze, 2015) avoiding the burden of surgery for patients whose condition worsens or progresses quickly during neoadjuvant therapy. However, there is no completed phase III clinical trial that proves the effectiveness of neoadjuvant therapy. One ongoing phase III clinical trial (GAIN trial) aims to verify the advantages of neoadjuvant therapy (3 cycles of gemcitabine combined with cisplatin (GC regimen) before and after surgery, followed by surgery) over surgery ± adjuvant therapy regimen in OS. The research subjects are pathologically confirmed pT2-3N- or pT1-3N + GBC patients after cholecystectomy, and the main results will be announced in 2024 (Goetze et al., 2020). Another ongoing phase III RCT (POLCAGB study) compares the efficacy of neoadjuvant chemoradiotherapy with neoadjuvant chemotherapy and verifies whether neoadjuvant chemoradiotherapy is superior in tumor downstaging and OS improvement (Engineer et al., 2019). The research subjects are patients with locally advanced T3/4 GBC confirmed by histopathology, who are randomly assigned to the chemotherapy group and chemoradiotherapy group based on the gemcitabine regimen (GC regimen or GEMOX regimen). The primary endpoint of this study is to compare the OS between the two groups of patients, and the secondary endpoint is to compare the progression-free survival (PFS) and R0 resection rate between them. The results of this trial are expected. In addition, the following issues in the process of neoadjuvant therapy should be fully explored: (Hundal and Shaffer, 2014): the timing and duration of neoadjuvant therapy, and (Sharma et al., 2017) the safety of large-scale surgery after neoadjuvant therapy, such as extended hepatectomy and/or pancreaticoduodenectomy.

## 4 Postoperative adjuvant therapy of GBC

In 2019, the PRODIGE12-ACCORD18 study reported that for patients with biliary tract cancer (BTC), postoperative adjuvant chemotherapy using the GEMOX regimen (gemcitabine + oxaliplatin) did not prolong the PFS compared with simple surgery (median PFS: 30.4 months vs. 18.5 months, hazard ratio (HR) = 0.88, *p* = 0.48) (Edeline et al., 2019). Subsequently, the OS of patients who received postoperative monotherapy (capecitabine) and those who underwent simple surgery was compared in the BILCAP study. According to the results, although the intention-to-treat analysis confirmed no statistically significant difference in the OS between the two groups (51.1 months vs. 36.4 months, HR = 0.81, *p* = 0.097), the per-protocol analysis revealed that the capecitabine therapy was more beneficial for the OS (53 months vs. 36 months, HR = 0.75, *p* = 0.028), and the capecitabine group showed high safety and tolerability (Primrose et al., 2019). Therefore, the guidelines of the American Society of Clinical Oncology (ASCO) and the National Comprehensive Cancer Network (NCCN) both recommend a 6-month capecitabine adjuvant therapy for patients with BTC (Shroff et al., 2019a; Benson et al., 2021). This regimen is also widely recognized as the standard treatment regimen after BTC resection worldwide. Additionally, many researchers are evaluating the efficacy of postoperative adjuvant radiotherapy and chemoradiotherapy (Wang et al., 2015; Kim et al., 2018). Horgan et al. (2012) analyzed the efficacy of adjuvant chemotherapy, radiotherapy and chemoradiotherapy in patients after BTC resection, and the results revealed that postoperative adjuvant therapy might be beneficial for BTC patients with positive resection margins (R1) and/or positive lymph nodes. In a multicenter retrospective study (Kim et al., 2016), the effect of radiotherapy on the prognosis of GBC patients after cholecystectomy was analyzed, which also demonstrated that postoperative adjuvant radiotherapy exerted the greatest effect on the improvement of the prognosis of patients with high-risk features [T3-4 tumor, positive lymph nodes (N1) and positive surgical margins (R1)]. However, different views appear recently, that is, adjuvant radiotherapy can also improve the prognosis of GBC patients with negative margins and negative lymph nodes after surgery (Kamarajah et al., 2022). In summary, many studies have shown that adjuvant radiotherapy and adjuvant chemoradiotherapy can improve the long-term survival rate after cholecystectomy (Gkika et al., 2020). Nonetheless, clinicians should cautiously interpret the conclusions of these studies since most of them are drawn based on retrospective research or SEER database analysis. In addition, there is an urgent need for prospective trials to prove the role of adjuvant radiotherapy or chemoradiotherapy. In a phase II prospective single-arm study (SWOG-0809), GBC patients with high risk (pT2-4, positive lymph nodes or positive surgical margins) received four cycles of gemcitabine plus capecitabine therapy (21-day regimen), followed by radiotherapy (the radiation dose to the regional lymph node was 45 Gy, and the radiation dose to the tumor bed was 54–59.4 Gy). The 2-year overall survival rate of the patients was 65%, considerably exceeding the expected effective threshold (assuming that the 2-year OS rate was >45%, the estimated total survival rate in R0 group was ≥65%, and the estimated total survival rate in R1 group was >45%) in 67% of patients in R0 resection group and 60% in R1 resection group (Ben-Josef et al., 2015). As the patients showed shows good tolerance and efficacy, the SWOG-0809 regimen is recommended by the ASCO and NCCN guidelines as a postoperative adjuvant therapy for GBC with R1 surgical margins (Benson et al., 2021). The ongoing phase III clinical trials (ACTICCA-1, NCT02170090) explore the efficacy of adjuvant therapy using the GC regimen. The monotherapy regimen (capecitabine) with a treatment cycle of 24 weeks is adopted in control group. A total of 781 patients with BTC (including hilar cholangiocarcinoma, distal cholangiocarcinoma, intrahepatic cholangiocarcinoma and GBC) after surgical resection were recruited in this trial, and disease-free survival (DFS) is the primary research endpoint (Stein et al., 2015). In the AdBTC-1 study (NCT03779035) in China, 460 patients with BTC (including GBC) after radical resection will be recruited and randomly assigned to gemcitabine plus capecitabine regimen group or capecitabine monotherapy group, with recurrence-free survival (RFS) as the primary endpoint. The results of the above two trials are worth our expectations. However, at present, adjuvant therapy after GBC resection still faces with some basic problems. For example, the BILCAP study has shown the advantages of postoperative adjuvant therapy in improving the survival of patients, but it has not evaluated the problem that patients have poor tolerance to adjuvant therapy due to impaired liver function after extensive hepatectomy or decreased physical status scores caused by surgery. These patients may not benefit from postoperative adjuvant therapy, but many studies have not mentioned the proportion and number of such patients, so further research is needed.

## 5 Unresectable advanced GBC

### 5.1 First-line treatment

Although the results of the ABC-02 phase III clinical trial have been published for over 10 years, the GC regimen (gemcitabine combined with cisplatin) remains the standard first-line treatment for patients with advanced unresectable or metastatic BTC (Valle et al., 2010). As nephrotoxicity is more common in the GC regimen, cisplatin can be replaced by oxaliplatin (Fiteni et al., 2014), but the only prospective phase III clinical study does not show the equivalent efficacy of gemcitabine combined with oxaliplatin (Sharma et al., 2019). Another phase III non-inferiority clinical trial demonstrated that the XELOX regimen (capecitabine + oxaliplatin) was not inferior to the GEMOX regimen in improving the 6-month PFS rate of patients (46.7% vs. 44.6%) (Kim et al., 2019). Moreover, many studies have made attempts to intensify the treatment with multidrug regimens. As revealed by the results of a Japanese study, the efficacy of gemcitabine combined with S-1 (GS regimen) is not inferior to that of the GC regimen. S-1 is an oral fluoropyrimidine drug composed of tegafur, gimeraci and oteracil potassium. The median OS was 13.4 months in GC group and 15.1 months in GS group (HR = 0.945, *p* = 0.046). In addition, hydration is not required by the GS regimen, but over 2 h of hydration is needed by the GC regimen to prevent cisplatin-induced nephrotoxicity (Morizane et al., 2019). Therefore, the GS regimen can be adopted as another first-line standard treatment regimen. Another phase III clinical trial comparing the GC regimen with the GCS regimen (cisplatin + gemcitabine + S-1) ascertained that the OS of the GCS regimen was longer than that of the GC regimen (13.5 months vs. 12.6 months, HR = 0.791, *p* = 0.046). In addition, the objective response rate (ORR) of the GCS regimen tripled that of the GC regimen (41.5% vs. 15.0%, *p* < 0.001). On this basis, GCS drugs are being tested in a phase III clinical trial (JCOG 1920) as a neoadjuvant therapy for patients with potentially resectable BTC (Ioka et al., 2023). However, the main drawbacks of the above two studies are that the target subjects are ethnically homogeneous, and only Japanese patients are enrolled, lacking international standardized results. The recent PRODIGE38-AMEBICA phase II clinical trial (NCT02591030) evaluated the efficacy of mFOLFIRINOX [5-fluorouracil (5-FU) + irinotecan + oxaliplatin] regimen and GC regimen, and it was found that no statistically significant differences in the median PFS and OS were detected between the two groups (Phelip et al., 2022). Furthermore, the ORR was not significantly improved by intensive therapy. The ORR was 25.0% in mFOLFIRINOX group and 19.4% in GC group, which was slightly lower than that in the GC group reported by ABC-02 (26.1%). As a result, the mFOLFIRINOX regimen is unlikely to be recommended as the first-line neoadjuvant therapy for patients with borderline resectable BTC. Shroff et al. (2019b) added nab-paclitaxel to the standard dual therapy (GC regimen), which prolonged the median PFS and OS of patients. The ORR of this regimen was 45%, the median PFS was 11.8 months (8 months in the ABC-02 study) and the median OS was 19.2 months (11.7 months in the ABC-02 study). However, the poor tolerance of patients to this regimen and the 20% reduction in the doses of gemcitabine and nab-paclitaxel compared with the standard therapeutic doses weakened the actual effect of this regimen to some extent. The ongoing phase III SWOG1815 trial (NCT03768414) evaluates the efficacy of the nab-paclitaxel + GC triple regimen with a modified dose in contrast to the GC regimen, and the conclusion is yet to be published.

### 5.2 Second-line treatment

Research has revealed that 15%–25% of patients whose tumor has progressed after first-line treatment can still receive second-line chemotherapy (Lamarca et al., 2014). In the ABC-06 phase III clinical trial, among the patients whose tumor had progressed after the first-line GC regimen, the efficacy of active symptom control + FOLFOX regimen (oxaliplatin + calcium folinate + fluorouracil) was compared with that of active symptom control alone. The results showed that the median OS in active symptom control + FOLFOX group was 6.2 months, while that in active symptom control group was 5.3 months (HR = 0.69, *p* = 0.031). The 6-month and 12-month OS rates in active symptom control group were 35.5% and 11.4%, respectively, while those in active symptom control + FOLFOX group were 50.6% and 25.9%, respectively (Lamarca et al., 2021). It should be noted that regardless of the patient’s previous sensitivity to platinum drugs, the efficacy of the FOLFOX regimen was maintained, with 30% of patients achieving at least 6 months of disease control. However, the limitation of this study is that the efficacy of the FOLFOX regimen was not compared with that of the monotherapy (fluorouracil) regimen, so it is not clear whether combination therapy is more effective than monotherapy (Tella et al., 2020). Irinotecan is a DNA topoisomerase I (Topo I) inhibitor. SN-38, a metabolite of irinotecan, can inhibit Topo I (a crucial substance involved in synthesizing DNA), which prevents DNA synthesis of tumor cells and exerts an anti-tumor effect. At present, SN-38 is mainly used for treating gastrointestinal tumors (Saltz et al., 2000; Pozzo et al., 2004). Several studies evaluated the potential efficacy of irinotecan as monotherapy or in combination with other drugs in BTC, which also confirmed the good anti-tumor activity and safety of irinotecan. In a phase II clinical study, whether mFOLFIRI (irinotecan + 5-FU) is superior to mFOLFOX (oxaliplatin + 5-FU) as the second-line treatment of BTC was determined (Choi et al., 2021). The results showed that mFOLFIRI was not superior to mFOLFOX. Specifically, the 6-month OS rate of patients in mFOLFOX group was 54.1%, while that in mFOLFIRI group was 44.1% (*p* = 0.677). Besides, the median OS in mFOLFOX group and mFOLFIRI group was 6.3 months and 5.7 months, respectively (*p* = 0.974). In a phase II multicenter study (GB-SELECT) (Ramaswamy et al., 2021), the efficacy of the CAPIRI regimen (capecitabine + irinotecan) and IRI regimen (irinotecan alone) in patients with advanced GBC was compared. It was uncovered that among the GBC patients who had previously received first-line therapy (gemcitabine-based therapy) and had disease progression, there was no statistically significant difference in the 6-month OS rate between patients undergoing the CAPIRI regimen and those undergoing the IRI regimen. Irinotecan monotherapy should be the first-line therapy option for these patients. In another phase II single-center study (Zheng et al., 2018), the efficacy of the XELIRI regimen and irinotecan monotherapy in the treatment of advanced BTC patients who had disease progression after the GC regimen. According to the results, the XELIRI regimen was superior to irinotecan monotherapy in prolonging PFS (3.7 months vs. 2.4 months, *p* = 0.036). In the phase IIb study (NIFTY), the efficacy of liposome irinotecan + fluorouracil + calcium folinate regimen and fluorouracil + calcium folinate regimen in the treatment of metastatic BTC patients who had disease progression after the GC regimen was compared. It was discovered that the median PFS of liposome irinotecan + fluorouracil + calcium folinate was markedly longer than that of fluorouracil + calcium folinate therapy group (7.1 months vs. 1.4 months, HR = 0.56, *p* = 0.0019). The most common grade 3-4 adverse events in liposome irinotecan + fluorouracil + calcium folinate group were neutropenia (24%) and fatigue or asthenia (13%), reflecting its good safety and tolerability (Yoo et al., 2021). Hence, liposome irinotecan + fluorouracil + calcium folinate regimen can be used as a standard second-line treatment option. A list of clinical trials of chemotherapy that included GBC is given in Table 2.

**TABLE 2:** Clinical trials at different stages of chemotherapy that included GBC.

Trial	Year	Phase	Line of therapy	Patient number		Treatment regimen	mPFS/RFS (months)	mOS (months)	Hazard ratio; *p*-value (OS)
				GBC	Total BTC				
PRODIGE12 Edeline et al. (2019)	2019	Ⅲ	Postoperative	38 (20.0%)	194	GEMOX vs. Obs	30.4 vs. 18.5	75.8 vs. 50.8	HR = 1.08; *p* = 0.74
BILCAP Primrose et al. (2019)^a^	2019	Ⅲ	Postoperative	79 (17.6%)	447	Cap vs. Obs	25.9 vs. 17.4	53.0 vs. 30.0	HR = 0.75; *p* = 0.028
SWOGS0809 Ben-Josef et al. (2015)	2015	Ⅱ	Postoperative	25 (31.6%)	79	Gem + Cap + Radio	R_0_ = 35; R_1_ = 34	R_0_ = 26; R_1_ = 23	NA
ABC-02 Valle et al. (2010)^a^	2010	Ⅲ	First-line	149 (36.3%)	410	Gem + Cis vs. Gem	8.0 vs. 5.0	11.7 vs. 8.1	HR = 0.64; *p* < 0.001
Sharma et al. (2019)^a^	2019	Ⅲ	First-line	243 (100%)	243	GEMOX vs. Gem + Cis	5.0 vs. 4.0	9.0 vs. 8.3	HR = 0.78; *p* = 0.057
Kim et al. (2019)^a^	2019	Ⅲ	First-line	61 (27.5%)	222	XELOX vs. GEMOX	5.8 vs. 5.3	10.6 vs. 10.4	NA; *p* = 0.131
JCOG1113 Morizane et al. (2019)^a^	2019	Ⅲ	First-line	137 (38.7%)	354	Gem + S-1 vs. Gem + Cis	6.8 vs. 5.8	15.1 vs. 13.4	HR = 0.945; *p* = 0.046
KHBO1401 Ioka et al. (2023)^a^	2022	Ⅲ	First-line	82 (33.4%)	246	Gem + Cis + S-1 vs. Gem + Cis	7.4 vs. 5.5	13.5 vs. 12.6	HR = 0.748; *p* = 0.015
PRODIGE38-AMEBICA Phelip et al. (2022)	2022	Ⅱ	First-line	33 (17.3%)	190	mFOLFIRINOX vs. Gem + Cis	6.2 vs. 7.4	11.7 vs. 13.8	NA
Shroff et al. (2019b)	2019	Ⅱ	First-line	13 (21.6%)	60	Gem + Cis + nab-Pac	11.8	19.2	NA
ABC-06 Lamarca et al. (2021)^a^	2021	Ⅲ	Second-line	34 (21.0%)	162	FOLFOX + ASC vs. ASC	4.0 vs. NA	6.2 vs. 5.3	HR = 0.69; *p* = 0.031
Choi et al. (2021)	2021	Ⅱ	Second-line	35 (29.7%)	118	mFOLFIRI vs. mFOLFOX	2.1 vs. 2.8	5.7 vs. 6.3	NA; *p* = 0.677
GB-SELECT Ramaswamy et al. (2021)	2021	Ⅱ	Second-line	98 (100%)	98	CAPIRI vs. IRI	2.27 vs. 3.12	5.16 vs. 6.28	HR = 0.98; *p* = 0.930
Zheng et al. (2018)	2018	Ⅱ	Second-line	12 (20.0%)	60	XELIRI vs. IRI	3.7 vs. 2.4	10.1 vs. 7.3	HR = 0.63; *p* = 0.107
NIFTY Yoo et al. (2021)	2021	Ⅱ	Second-line	53 (30.5%)	174	5-FU + LVF + Nal-IRI vs. 5-FU + LVF	7.1 vs. 1.4	8.6 vs. 5.5	HR = 0.68; *p* = 0.035

^a^ Major Phase III clinical trials of medications that are commercially accessible.

Abbreviations: GBC, gallbladder cancer; BTC, biliary tract cancer; GEMOX, gemcitabine and oxaliplatin; Obs, observation; Cap, capecitabine; Gem, Gemcitabine; Cis, cisplatin; HR, hazard ratio; OS, overall survival; PFS, progression-free survival; RFS, recurrence free survival; Radio, Radiotherapy; NA, not achieved; S1, tegafur-gimeraci-oteracil potassium; XELOX, capecitabine and oxaliplatin; mFOLFIRINOX, modified FOLFIRINOX (5-fluorouracil and irinotecan and oxaliplatin); FOLFIRI, 5-fluorouracil and irinotecan; FOLFOX, 5-fluorouracil and leucovorin and oxaliplatin; nab-Pac, nanoliposomal-Paclitaxel; ASC, active supportive care; mFOLFOX, modified FOLFOX (oxaliplatin and 5-FU); mFOLFIRI, modified (FOLFIRI irinotecan and 5-FU); CAPIRI, capecitabine and irinotecan; IRI, irinotecan; XELIRI, capecitabine and irinotecan; 5-FU, 5-fluorouracil; Nal-IRI, nanoliposomal-irinotecan; LVF, leucovorin.

## 6 Targeted therapy and immunotherapy

With the development of advanced sequencing technologies including next-generation sequencing (NGS), whole exome sequencing (WES), RNA sequencing (RNAseq) and single-cell analysis, the characterization and thus global identification of genetic and epigenetic features and key molecules as potential therapeutic targets for gallbladder cancer, particularly in targeted therapies and immunotherapy (Voss et al., 2013). The molecular pathology of GBC is characterised by a high incidence of somatic mutations in the inactivated TP53 (tumor protein 53) gene. In a systematic evaluation based on 62 articles containing 3893 GBC samples, Kuipers et al. found that TP53 (tumor protein 53) was the most frequently mutated gene in approximately 57% (range 4%–71%) of all GBC patients (Kuipers et al., 2021b). Other common genetic changes in GBC patients are SHH (sonic hedgehog gene; about 20%), ELF3 (E74-like ETS transcription factor 3; about 18.6%), and ARID1A (AT-rich interactive domain-containing protein 1A; about 14%). SMAD4 (SMAD family member 4; about 13.1%), EGFR (epidermal growth factor receptor, synonym: Her 1, ERBB1; about 12%), ERBB2 (human epidermal growth factor receptor 2; synonym: her2/neu; about 10%), PIK3CA (Phosphorylinositol-4,5-Bisphosphate 3-Kinase Catalytic Subunit Alpha; about 14.6%), and KRAS (kirsten rat sarcoma virus; about 10.3%) (Javle et al., 2014; Javle et al., 2016; Roa et al., 2016; Dixit et al., 2017; Yang et al., 2019; Kuipers et al., 2021b).

### 6.1 EGFR and VEGF pathway

Epidermal growth factor receptor (EGFR) inhibitors and vascular endothelial growth factor receptor (VEGF) inhibitors are targeted drugs commonly used in antitumor treatment. EGFR is an essential transmembrane tyrosine kinase involved in activating the RAS/RAF/MAPK and AKT/mTOR signal transduction pathways, which are directly related to both cell proliferation and cell death (Jorissen et al., 2003). Both VEGF overexpression and micro-vessel density (MVD) have been put in correlation with cancer progression, metastasis and prognosis in GBC (Chen et al., 2011). VEGF is widely acknowledged to play an important role in promoting tumor angiogenesis. It has been shown that VEGF is highly expressed in the serum of GBC patients and promotes angiogenesis, proliferation and invasion of gallbladder cancer cells and inhibits apoptosis of tumor cells (Xu et al., 2019). Morizane et al. (2018) summarized the results of phase II clinical trials by adopting monotherapy or combination chemotherapy with targeted therapeutic drugs (erlotinib, cetuximab, panitumumab ± bevacizumab, sorafenib, cediranib, trametinib, and vandetanib), and believed that beneficial effects can be achieved by the GC regimen, GEMOX regimen or monotherapy (gemcitabine) regimen combined with targeted drugs in the treatment of BTC patients. However, the effectiveness of EGFR inhibitors and VEGF inhibitors, either used alone or in combination with chemotherapy, is basically the same as that of the standard first-line treatment regimen. The above conclusion is confirmed by a meta-analysis. Rizzo et al. (2020) analyzed 4 related phase II clinical trials to explore the efficacy of EGFR inhibitors (two cetuximab regimens and two panitumumab regimens) and first-line chemotherapy based on the gemcitabine regimen (three GEMOX regimens and one GC regimen) in the treatment of patients with advanced BTC. It was found that the combination with anti-EGFR monoclonal antibody did not improve ORR, PFS or OS in patients receiving first-line treatment. Nevertheless, the single or combined use of targeted drugs plays a good role in the second-line treatment of advanced BTC. In a randomized, double-blind, phase II clinical trial (REACHIN) (Demols et al., 2020), the safety and efficacy of regorafenib in unresectable or metastatic BTC patients who had disease progression after chemotherapy using gemcitabine combined with platinum. As demonstrated by the results, the median PFS in regorafenib group was remarkably longer than that in placebo group (3.0 months vs. 1.5 months, *p* = 0.004), and the 6-month PFS rate in regorafenib group was larger than that in placebo group (21% vs. 3%). A phase II single-arm trial was performed to assess the efficacy of ramucirumab (a targeted VEGFR-2 drug), in the treatment of advanced BTC patients who had previously received gemcitabine-based chemotherapy (Lee et al., 2022). The results manifested that the median PFS and OS were 3.2 months and 9.5 months, respectively, and PFS was similar to that of other chemotherapy regimens previously used for refractory BTC.

### 6.2 HER2/neu pathway

HER2 is a cell-surface receptor with a transmembrane tyrosine kinase domain that plays a crucial role in tumor biology through the downstream activation of the PI3K/Akt pathway (call polarity, cell adhesion, cell cycle) and the MAPK pathway (mitosis) in multiple cancers (Roskoski, 2014; Yan et al., 2014). In GBC, HER2/neu overexpression is more common than in other forms of BTC (Kiguchi et al., 2001). Blocking human epidermal growth factor receptor 2 (HER-2) is therefore an alternative strategy for the targeted treatment of GBC (Roa et al., 2014). In the TreeTopp study, the role of varlitinib in advanced BTC patients was evaluated (Javle et al., 2022), and it was found that patients showed high tolerance to the second-line treatment with varlitinib + capecitabine, but it did not improve the efficacy compared with capecitabine alone. However, the median PFS was prolonged in female patients and GBC patients. In the MyPathway basket study (Javle et al., 2021), 39 patients with metastatic BTC (16 patients with GBC) had been previously treated and had HER2 amplification, overexpression or both. Nine patients achieved partial response (PR) (the ORRs of BTC and GBC were 23% and 31%, respectively), and the median duration was 10.8 months. The median PFS and OS were 4 months and 10.8 months, respectively. In another basket trial in which patients with HER2 and HER3 mutations were treated with neratinib, 3 of 25 patients with BTC had a PR, and the ORR was 16%, proving that anti-HER2 monoclonal antibodies have a certain effect on specific advanced GBC patients (Harding et al., 2022). Zanidatamab is a bispecific HER2-targeting antibody, which has been proven to have antitumor efficacy and safety in HER2-overexpressing cancers. In a phase I study enrolling 20 patients with unresectable, locally advanced or metastatic BTC (11 patients with GBC) that had progressed after first-line therapy, the ORR and disease control rate (DCR) were 47% and 65%, respectively, with a median response duration of 6.6 months after treatment with zanidatamab, and it showed good safety and tolerance (Meric-Bernstam et al., 2021). Based on these data, zanidatamab is being assessed in an ongoing global phase IIb study (NCT04466891) in advanced HER2-positive BTC patients who have disease progression after gemcitabine-containing regimens. Additionally, three ongoing trials (NCT03613168, NCT02992340, NCT02836847) are designed to evaluate the role of anti-HER2 targeted therapy in BTC patients, and the results are prospective. Moreover, PIK3CA mutations are present in some GBC patients, which activate the PI3K/Akt/mTOR pathway and promote the occurrence and development of GBC (Roa et al., 2016). In the phase II study of everolimus monotherapy as first- and second-line therapy for advanced BTC, the ORR was reduced from 12% in the first-line treatment to 5.1% in the second-line treatment. Moreover, the DCR of GBC is obviously worse as compared to that of other BTCs (Buzzoni et al., 2014; Lau et al., 2018).

### 6.3 PD-1/PD-L1 pathway

The immune microenvironment of BTC has been shown to be suppressive with decreased cytotoxic immune cells, increased T regulatory cells, and overexpression of PD1 and cytotoxic T lymphocyte-associated protein 4 (CTLA4) molecules on infiltrating T cells (Zhou et al., 2019). Programmed death-1 (PD-1), are frequently exploited through overactivation by their specific ligands [e.g., programmed death ligand 1 (PD-L1)] that are expressed on cancer and immune cells, leading to peripheral T cell exhaustion, and thus allowing for tumor escape from immune surveillance (Fabris et al., 2019). In several clinical trials of immunotherapy, great achievements of pembrolizumab (anti-PD-L1) and nivolumab (anti-PD-1) have been made in GBC treatment. KEYNOTE-158 (Marabelle et al., 2020) and KEYNOTE-028 (Ott et al., 2019) studies aim to explore the efficacy and safety of pembrolizumab in the treatment of advanced cholangiocarcinoma. According to comprehensive research results, the ORR, median OS and PFS were 5.8% vs. 13.0%, 7.4 months vs. 5.7 months, and 2.0 months vs. 1.8 months, respectively (Piha-Paul et al., 2020). It can also be concluded that pembrolizumab ensures lasting anti-tumor activity in 6%–13% of patients with advanced BTC, regardless of the expression level of PD-L1, and it has controllable drug toxicity. The results of a phase II trial (Kim et al., 2020) revealed that all subjects showed good tolerance to nivolumab, with an ORR of 22%, a DCR of 59%, a median PFS of 3.68 months, and a median OS of 14.24 months. Additionally, immunotherapy combined with targeted drugs or chemotherapy drugs is also under clinical research. In another phase I trial (MakotoUeno), the efficacy of nivolumab monotherapy or nivolumab combined with the GC regimen in the treatment of patients with advanced BTC was explored. The median OS was 5.2 months vs. 15.4 months, and the median PFS was 1.4 months vs. 4.2 months, respectively, indicating that the efficacy of combined medication group is better than that of monotherapy group (Ueno et al., 2019). The latest results of the phase II multi-cohort study LEAP-005 demonstrated that the addition of lenvanib on the basis of pembrolizumab (anti-PD-1) is effective and well-tolerated in patients. In 31 BTC patients, the ORR and DCR were 10% and 68%, respectively (Villanueva et al., 2021). As revealed by the results of the phase II single-arm study REGOMUNE (Cousin et al., 2022), regorafenib combined with avelumab displayed anti-tumor activity in BTC patients who had been previously treated. Among 29 cases evaluable for efficacy, there were 4 cases (13.8%) of PR, but the primary endpoint of the experimental design was not reached. In another multicenter, phase II clinical study (NCT03092895) (Chen et al., 2021a), the feasibility of chemotherapy based on camrelizumab combined with oxaliplatin-based chemotherapy as a first-line treatment for advanced BTC was evaluated. The ORR and DCR were 16.3% and 75.0%, respectively, and the median PFS and OS were 5.3 months and 12.4 months, respectively, indicating the good efficacy and safety of this regimen. In a randomized phase II trial, the combination of the PD-L1 inhibitor atezolizumab and MEK inhibitor cobimetinib was applied in the treatment of advanced BTC. The results showed that in contrast to atezolizumab monotherapy, the combination of atezolizumab and cobimetinib reached its primary endpoint and remarkably prolonged the PFS (median PFS: 3.65 months vs. 1.87 months, HR = 0.58, *p* = 0.027) (Yarchoan et al., 2021). Recently, the results of the world’s first randomized, double-blind, placebo-controlled, global multicenter phase III clinical trial (TOPAZ-1) assessing the first-line immunotherapy (durvalumab) + GC regimen for advanced BTC have been announced, which is a milestone. The results showed that compared with chemotherapy alone, the combination with durvalumab evidently prolonged the OS of patients (median OS: 12.8 months vs. 11.5 months, HR = 0.80, *p* = 0.021) and PFS (median PFS: 7.2 months vs. 5.7 months, HR = 0.75, *p* = 0.001), and increased the ORR (26.7% vs. 18.7%), and the combined immunotherapy did not increase the incidence rate of grade 3-4 treatment-related adverse events (Oh et al., 2022). Another global, randomized, double-blind, multicenter phase II study (IMbrave151) is also under way to evaluate the role of bevacizumab + atezolizumab (anti-PD-L1) + gemcitabine + cisplatin as the first-line treatment regimen (Hack et al., 2021). Although only the preliminary results indicated that PD-1/PD-L1 inhibitors can be used for the treatment of advanced GBC, the positive results of TOPAZ-1 research mark the arrival of a new era of immunotherapy for advanced GBC, and it is expected to change the treatment mode of GBC patients. Table 3 summarizes main clinical trials of targeted therapy and/or immunotherapy including GBC.

**TABLE 3:** Main clinical trials of targeted therapy and/or immunotherapy including gallbladder cancer.

Trial	Year	Phase	Target	Line of therapy	Patient number		Treatment regimen	mPFS (months)	mOS (months)	ORR (%)
					GBC	BTC				
Lee et al. (2012)	2012	Ⅲ	EGFR	First line	82	268	GEMOX ± erlotinib vs. GEMOX	5.8 vs. 4.2	9.5 vs. 9.5	40 vs. 21
REACHIN Demols et al. (2020)	2020	Ⅱ	VEGF and multiple other targets	Second line	9 (13.6%)	66	Regorafenib	3	5.3	NA
Lee et al. (2022)	2022	Ⅱ	VEGF-2	Second line	13 (21.7%)	60	Ramucirumab	3.2	9.5	NA
MyPathway Javle et al. (2021)	2021	Ⅱ	HER2/neu	Second line	16 (41.0%)	39	Pertuzumab + trastuzumab	4	10.9	23
TreeTopp Javle et al. (2022)	2022	Ⅱ	HER1, HER2 and HER4	Second line	34 (26.8%)	127	Varlitinib + cap vs. cap	2.83 vs. 2.79	7.8 vs. 7.5	9.4 vs. 4.8
James Harding et al. (2022)	2022	Ⅱ	HER2, HER3	Second line	10 (40%)	25	Neratinib	2.8	5.4	16
ZW25 Meric-Bernstam et al. (2021)	2021	I	HER2	Second line	11 (44%)	20	Zanidatamab	NA	NA	47
RADiChol Lau et al. (2018)	2018	Ⅱ	mTOR	First line	12 (44%)	27	Everolimus	5.5	9.5	12
KEYNOTE-158 Marabelle et al. (2020)	2020	Ⅱ	PD-1	Second or later	NA	104	Pembrolizumab	2	9.1	5.8
Kim et al. (2020)	2020	Ⅱ	PD-1	Second or later	17 (31%)	54	Nivolumab	3.7	12.4	22
MakotoUeno Ueno et al. (2019)	2019	I	PD-1	First line	20 (33%)	60	Nivolumab + Gem + Cis vs. Nivolumab	4.2 vs. 1.4	15.4 vs. 5.2	11 vs. 1
LEAP-005 Villanueva et al. (2021)	2021	Ⅱ	PD-1 and multiple other targets	Second or later	NA	31	Lenvatinib + Pembrolizumab	6.1	8.6	NA
REGOMUNE Cousin et al. (2022)	2022	Ⅱ	PD-L1 and multiple other targets	Second or later	1 (2.9%)	34	Regorafenib + avelumab	2.5	11.9	NA
Chen et al. (2021a)	2021	Ⅱ	PD-1	First line	11 (12%)	92	Camrelizumab + GEMOX or FOLFOX	5.3	12.4	16.3
Yarchoan et al. (2021)	2021	Ⅱ	PD-L1, MEK	Second or later	19 (24.7%)	77	Atezolizumab ± cobimetinib	3.56 vs. 1.87	NA	1 vs. 1
TOPAZ-1 Oh et al. (2022)^a^	2022	Ⅲ	PD-L1	First line	171 (24.9%)	685	Durvalumab + Gem + Cis vs. Gem + Cis	7.2 vs. 5.7	12.8 vs. 11.5	26.7 vs. 18.7

^a^ Major Phase III clinical trials of medications that are commercially accessible.

Abbreviations: GBC, gallbladder cancer; BTC, biliary tract cancer; GEMOX, gemcitabine and oxaliplatin; PFS, progression-free survival; OS, overall survival; ORR, objective response rate; EGFR, epidermal growth factor receptor; VEGF, vascular endothelial growth factor; HER, herceptin receptor; PD-1, programmed death 1; PD-L1, programmed death ligand1. MEK, MAPK/ERK kinase. NA, not achieved; FOLFOX, 5-fluorouracil and leucovorin and oxaliplatin; Cap, capecitabine; Gem, Gemcitabine; Cis, cisplatin; mTOR, mammalian target of rapamycin.

## 7 Challenges and future prospects

Obviously, it should be pointed out that the above comprehensive treatment options for GBC, especially the formulation of drug therapy regimens, are mostly introduced from the research data of biliary tract tumors. In addition, most of the current studies on drug therapy for biliary tract tumors usually involve GBC and cholangiocarcinoma. There has been evidence from multiple dimensions such as molecular biology (Chen et al., 2021b; Yoon et al., 2021) and clinical data (Azizi et al., 2021) that GBC and cholangiocarcinoma not only differ in anatomy, but also in disease behaviors, molecular characteristics and sensitivity to treatment, and their responses to the same treatment regimen also show large differences. However, there are few prospective clinical trials on GBC alone. Attention should be paid to the differences between GBC and other biliary tract tumors in the future. Additionally, more GBC patients should be enrolled, and multi-center institutional collaboration and standardization should increase the quality of clinical studies. More precise information on the therapy of GBC can only be supplied by adopting high-quality clinical research or more rigorous subgroup analysis. Ongoing trials enrolling patients with GBC are listed in Table 4.

**TABLE 4:** Ongoing trials enrolling patients with gallbladder cancer-clinical trial information obtained from ClinicalTrials.gov.

Identifier	Phase	Line of therapy	Treatment regimen	Population	Primary EndPoint	Status
NCT03673072	III	Neoadjuvant	Perioperative Gem + Cis vs. adjuvant	Incidental GBC and BTC	OS	Recruiting
NCT02867865	II/III	Neoadjuvant	RT + Gem + Cis vs. Gem + Cis	GBC	OS	Recruiting
NCT04308174	II	Neoadjuvant	Durvalumab + Gem + Cis vs. Gem + Cis	BTC (including GBC)	R0 resection rate	Recruiting
NCT04559139	II/III	Neoadjuvant	Perioperative Gem + Cis vs. adjuvant	GBC	OS	Recruiting
NCT05036798	II	Neoadjuvant	Tislelizumab + Lenvatinib + Gemox	BTC (including GBC)	R0 resection rate	Active, not recruiting
JCOG1920	III	Neoadjuvant	Gem + S-1 vs. Gem + Cis + S-1	BTC (including GBC)	OS	Recruiting
NCT03779035	II	Postoperative	Gem + Cap vs. Cap	BTC (including GBC)	DFS rate 2 yr	Recruiting
NCT05254847	II	Postoperative	Cap + Lenvatinib + Tislelizumab	BTC (including GBC)	DFS rate 1 yr	Recruiting
NCT03768414	Ⅲ	First line	Gem + Cis + nab-Pac vs. Gem + Cis	BTC (including GBC)	OS	Recruiting
NCT04692051	II	First line	nab-Pac + Cis vs. Gem + Cis	BTC (including GBC)	PFS	Unknown
NCT04203160	IB/II	First line	Devimistat + Gem + Cis vs. Gem + Cis	BTC (including GBC)	DLT, ORR	Recruiting
NCT04003636	Ⅲ	First line	Pembrolizumab + Gem + Cis vs. GEM + Cis	BTC (including GBC)	OS	Active, not recruiting
NCT03473574	Ⅱ	First-line	Durvalumab + Tremelimumab + Gem/Gem + Cis vs. Gem + Cis	GBC and CCA	ORR	Active, not recruiting
NCT02711553	Ⅱ	First-line	Ramucirumab or Merestinib or Placebo + Gem + Cis	BTC (including GBC)	PFS	Active, not recruiting
NCT04191343	Ⅱ	First-line	Toripalimab + GEMOX	BTC (including GBC)	ORR	Recruiting
NCT04300959	Ⅱ	First-line	Anlotinib hydrochloride + PD1 + Gem + Cis vs. Gem + Cis	BTC (including GBC)	OS 1 yr	Unknown
NCT04677504	Ⅱ	First-line	Atezolizumab + Bevacizumab + Gem + Cis vs. Gem + Cis	BTC (including GBC)	PFS	Active, not recruiting
NCT03478488	Ⅲ	First line	Envafolimab + gem + oxaliplatin	BTC (including GBC)	OS	Recruiting
NCT04720131	Ⅱ	First line	Camrelizumab + apatinib + capecitabine	BTC (including GBC)	ORR	Not yet recruiting
NCT03796429	Ⅱ	First line	Toripalimab + gem + S-1	BTC (including GBC)	PFS, OS	Recruiting
NCT03043547	Ⅱ	Second or later	Nal-IRI + 5-FU vs. 5-FU	GBC and CCA	PFS	Active, not recruiting
NCT04722133	II	Second or later	Trastuzumab-pkrb + mFOLFOX6	BTC (including GBC)	ORR	Recruiting
NCT04306367	II	Second or later	Pembrolizumab + Olaparib	BTC (including GBC)	ORR	Unknown
NCT03639935	II	Second or later	Rucaparib + Nivolumab	BTC (including GBC)	4-month PFS rate	Active, not recruiting
NCT03785873	Ib/II	Second-line	Nal-IRI + Nivolumab + 5-FU + Leucovori	BTC (including GBC)	DLT, PFS	Active, not recruiting
NCT04781192	I/II	Second or later	Regorafenib + durvalumab	BTC (including GBC)	AEs, PFS	Recruiting
NCT03639935	II	Second or later	Rucaparib + Nivolumab	BTC (including GBC)	PFS	Active, not recruiting
NCT04781192	I/II	Second or later	Regorafenib + Durvalumab	BTC (including GBC)	AEs, PFS	Recruiting
NCT04057365	II	Second or later	DKN-01 + Nivolumab	BTC (including GBC)	ORR	Recruiting
NCT05170438	II	Second or later	Lenvatinib + Paclitaxel	BTC (including GBC)	ORR	Recruiting

Abbreviations: GBC, gallbladder cancer; BTC, biliary tract cancer; Cap, capecitabine; Gem, Gemcitabine; Cis, cisplatin; GEMOX, gemcitabine and oxaliplatin; S-1, tegafur-gimeraci-oteracil potassium; DLT, dose-limiting toxicity; PFS, progression-free survival; DFS, disease free survival; ORR, objective response rate; RT, radiotherapy; Nab-pac, nab-paclitaxel; Pac, paclitaxel; 5-FU, 5-fluorouracil; Nal-IRI, nanoliposomal-irinotecan; AEs, adverse events; OS, overall survival; mFOLFOX6, 5-fluorouracil and leucovorin and oxaliplatin.

## 8 Conclusion

Due to high invasiveness and tumor heterogeneity, the treatment of GBC is still faced with severe challenges. Standardized radical operation at the early stage and comprehensive treatment based on chemotherapy at the advanced stage are still standard methods for the treatment of GBC. With the continuous deepening of research, the current treatment of GBC has changed tremendously. Treatment medications are no longer restricted to chemotherapy or circumscribed therapy, and more and more research is focusing on the use of combination regimens. Cytotoxic chemotherapy’s mechanism of action, and the main driver of its anticancer effect, is its ability to damage DNA during cellular division. Increasingly, the immunogenic effect of cytotoxic treatment is becoming evident with the advent of effective immune checkpoint blockade. Likewise, multitargeted TKIs and VEGF inhibition, which may promote T-cell activation and reduce immunosuppressive regulatory T cells in tumors *in vivo*, may enhance the effect of anti-PD-1 therapy. The prognosis of patients with GBC in palliative care and good ECOG status may be improved by exploiting potential synergies to enhance treatment, such as using a combination of three or four chemotherapy regimens. We must also be aware that next-generation sequencing tests are imperative for all newly diagnosed patients with advanced GBC, and that molecular genetic analysis and related biomarker studies (e.g., gene drivers, tumor mutational burden status, inflamed cytotoxic T-cell score, immune microenvironment composition, and host microbiome) will help to develop and evaluate effective drugs, address resistance mechanisms and manage drug-related toxic effects, as well as improve patient prognosis and maintain an efficient quality of life.
